# Effect of Switching Therapy to Pegaptanib in Eyes With the Persistent Cases of Exudative Age-Related Macular Degeneration

**DOI:** 10.1097/MD.0000000000000116

**Published:** 2014-10-10

**Authors:** Chieko Shiragami, Aoi Ono, Mamoru Kobayashi, Saki Manabe, Ayana Yamashita, Fumio Shiraga

**Affiliations:** Department of Ophthalmology (CS, AO, MK, SM, AY), Kagawa University Faculty of Medicine, Kagawa; and Department of Ophthalmology (FS), Okayama University Medical School, Okayama, Japan.

## Abstract

Purpose of this study was to evaluate the efficacy of switching to pegaptanib monotherapy for persistent cases of exudative age-related macular degeneration (AMD).

Out of 296 eyes of 296 patients treated with ranibizumab or ranibizumab combined with photodynamic therapy (PDT), 50 eyes of 50 AMD patients were found to be resistant to these treatments. Over a 12-month period, intravitreal pegaptanib (IVP) 0.3 mg was administered at intervals of 6 weeks until the exudation disappeared prospectively. All patients were examined with the following tests: best-corrected visual acuity (BCVA) and central retinal thickness (CRT), determined at the initial visit, before the first IVP (baseline), and at 12 months. The factors responsible for achieving dry macula with IVP were examined statistically.

The rate of persistent cases with intravitreal ranibizumab (IVR) and/or PDT was 17.0%. The mean number of IVPs administered was 5.4 (range, 2–9). Logarithm of the minimal angle of resolution BCVA at 12 months was stable or improved by ≥0.3 in 49 eyes (98.0%), with a significant improvement noted between the baseline and final BCVA (*P* = 0.01, paired *t* test). The CRT (mean ± standard deviation) was 446.9 ± 150.6 µm at the initial visit, 414.5 ± 146.5 µm at baseline, and 318.7 ± 99.0 µm at 12 months. There was a significant decrease in the mean CRT between the measurements at baseline and at 12 months after the first IVP (*P* = 0.002, Bonferroni correction). At 12 months, the exudative change was completely resolved in 27 eyes (54.0%) and reduced in 21 eyes (42.0%). The number of previous IVR treatments was significantly correlated with dry macula at 12 months.

After switching therapy to pegaptanib in persistent cases of AMD, most patients maintained or improved their BCVA and exhibited a positive treatment response at 12 months.

## INTRODUCTION

Intravitreal agents have been used to clinically treat exudative age-related macular degeneration (AMD). Agents used include pegaptanib sodium (Macugen; Valeant Pharmaceuticals/Pfizer Inc, New York, NY), which is an RNA aptamer that targets vascular endothelial growth factor^1,2^ (VEGF)_165_, and ranibizumab (Lucentis; Genentech Inc, South San Francisco, CA), which is a monoclonal antibody fragment that binds with all VEGF-A isoforms over a period of several years.^3,4^ In pivotal trials, ranibizumab was shown to provide significant improvement of the mean visual acuity as compared with the control therapy for AMD.^3,4^ It has been suggested that these results may be related to a mechanism of action in which ranibizumab binds to all VEGF-A isoforms.^3–6^

Even though ranibizumab is usually administered for AMD, it is not uncommon for patients receiving this treatment to frequently develop tachyphylaxis.^7,8^ Keane et al^9^ first reported finding possible tachyphylaxis after AMD treatments. Their report concluded that even though the neurosensory retinal edema and subretinal fluid showed an early reduction to nadir after intravitreal ranibizumab (IVR) therapy, there was attenuation of the effect on the retina over time, which suggested possible tachyphylaxis. Several published studies have demonstrated that aflibercept was effective for reducing the exudation that resulted in tachyphylaxis due to ranibizumab during the treatment of wet AMD.^10–12^ These studies additionally reported that switching the therapy to aflibercept was only effective for reducing exudation and decreasing the central retinal thickness (CRT). This change in therapy did not lead to an improvement in the visual function.

The most important endpoint for wet AMD treatment should be not only the improvement or long-term maintenance of the visual function, but also the reduction of the exudation. The selection of further treatment depends on many factors, including the activity of recurrent choroidal neovascularization (CNV), presence of geographic atrophy (GA), and prior treatments, as well as individual patient considerations. Up until now, there have been no reports that have evaluated switching persistent wet AMD patients to pegaptanib monotherapy from other anti-VEGF agents. Therefore, the aim of the current study was to investigate the outcome and effect of switching persistent AMD cases resistant to ranibizumab, and/or combined photodynamic therapy (PDT), to pegaptanib monotherapy.

## PATIENTS AND METHODS

Between April 2010 and April 2012, we prospectively reviewed 296 eyes of 296 patients treated with ranibizumab or ranibizumab combined with PDT. A total of 50 eyes of 50 AMD patients were found to be resistant to these treatments.

Before starting pegaptanib monotherapy, all 296 patients received 3 initial consecutive monthly IVR injections followed by pro re nata. PDT-combined therapy with 3 monthly loading doses was performed for most of the polypoidal choroidal vasculopathy (PCV) and retinal angiomatous proliferation (RAP) patients.

Despite an initial good response to the IVR treatment, optical coherence tomography (OCT) showed thickening of the macular exudate, and there was deterioration of the visual function in 50 of the patients. Over a 12-month period, intravitreal pegaptanib (IVP) 0.3 mg was administered at 6 week intervals until the exudative lesions resolved. Because there were not so many persistent AMD cases resistant to ranibizumab and/or combined PDT in this case series, all patients were examined for the effect of switching to pegaptanib monotherapy without establishing the control group.

All patients were examined with the following tests: best-corrected visual acuity (BCVA), fundus color photography and fluorescein angiography (FA) using the Topcon TRC-50DX fundus camera (Topcon, Tokyo, Japan), indocyanine green angiography (ICGA), and OCT using a Spectralis/Heidelberg Retina Angiograph 2 (Heidelberg Engineering, Heidelberg, Germany), at the initial visit, baseline, and at every subsequent visit during the 12-month follow-up period. CRT between the inner limiting membrane and Bruch membrane was measured by Spectralis. Lesion type, location, and activity of the CNV at the initial visit were determined using FA and ICGA.

During the study period, OCT was used to monitor the resolution and recurrence of fluid in the eyes as the pegaptanib therapy was started and stopped. Patients were usually treated when OCT imaging indicated evidence of recurrent fluid.

Statistical analyses were performed to compare the BCVA at the initial visit and immediately before the first IVP (baseline) with the BCVA at 12 months (paired *t* test). Analyses were also performed to compare the CRT between the initial visit, baseline, and at 12 months (Bonferroni correction). Moreover, the CRT was compared between baseline and 12 month for each type of AMD (paired *t* test). Multiple logistic regression analysis was performed in order to determine whether there was a significant association between the independent variables (which included age, previous treatments, and duration of disease) and the dependent variable (achieving dry macula at 12 months after baseline). A *P* value <0.05 was considered significant except for Bonferroni correction. The statistical analyses were carried out using SPSS Statistics version 21 (IBM SPSS, Inc, Chicago, IL).

This study was conducted in accordance with the Declaration of Helsinki. The study protocol and the subject-informed consent document were approved by the institutional review board/ethics committee at Kagawa University Faculty of Medicine. All patients gave their written, informed consent prior to participating.

## RESULTS

The clinical characteristics and clinical data for the 50 AMD patients who were switched to pegaptanib monotherapy are summarized in Table 1. The patients’ ages ranged from 61 to 90 years (mean, 77.7 years). Of the 50 AMD cases, 21 eyes were typical AMD (t-AMD), 23 eyes were PCV, and 6 eyes was RAP. FA findings at the initial visit were classified as occult with no classic lesions (7 eyes, 14.0%), minimally classic lesions (27 eyes, 54.0%), and predominantly classic lesions (16 eyes, 32.0%). Of the 296 eyes treated with IVR or PDT combined therapy, 50 eyes (16.9%) developed resistance after an initial positive morphological response to ranibizumab. On average, these patients each received a total of 7.1 ± 3.0 injections of ranibizumab (range, 4–14 injections) and underwent PDT for a total of 0.68 ± 0.65 times.

**Table 1:**
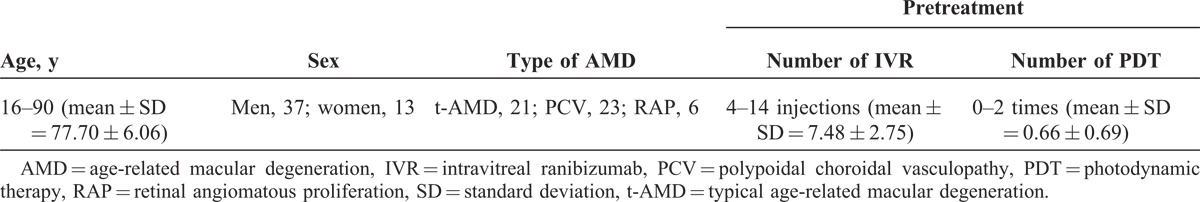
Baseline Clinical Characteristics of Persistent Cases of AMD in Patients Treated With Pegaptanib Monotherapy

### Visual Outcomes

**FIGURE 1:**
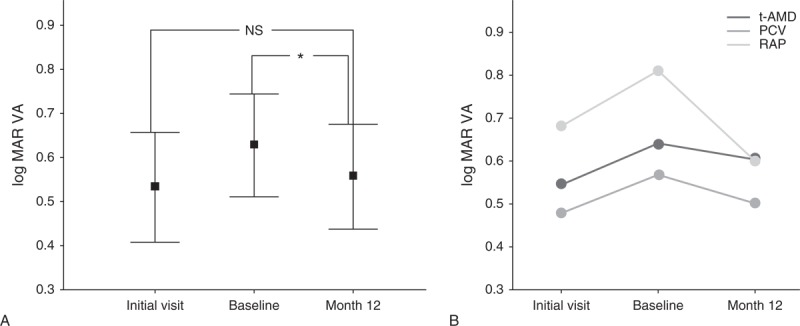
(A) Mean change in logMAR VA (whole type of AMD cases). This plot graph shows the changes in the mean BCVA in logMAR units at the initial visit, baseline, and at 12 months. The error bar values indicate the standard deviation. The logMAR BCVA (mean ± standard deviation) was 0.53 ± 0.44 at the initial visit, 0.63 ± 0.41 at baseline, and 0.56 ± 0.42 at 12 months. A significant improvement was seen between the baseline and the BCVA at the last visit (∗*P* = 0.01, paired *t* test). (B) Mean change in logMAR VA (each type of AMD cases).  
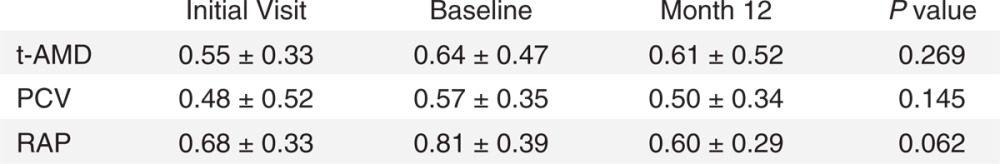

Logarithm of the minimal angle of resolution (logMAR) BCVA was improved by ≥0.3 in 9 eyes, stable in 40 eyes, and worsened by ≥0.3 in 1 eye at 12 months. The BCVA was stable or improved by ≥0.3 in 49 eyes (98.0%). As for the breakdown according to the type of AMD that logMAR BCVA improved by ≥0.3, there was 3 eyes (14.3%) in t-AMD, 4 eyes (17.4%) in PCV, and 2 eyes (50%) in RAP. The logMAR BCVA (mean ± standard deviation) was 0.53 ± 0.44 at the initial visit, 0.63 ± 0.41 at baseline and 0.56 ± 0.42 at 12 months. There was a significant improvement between the baseline and final BCVA (*P* = 0.011, paired *t* test) (Figure 1A).

*P* value indicates the statistical correlation of logMAR BCVA between baseline and 12 months (paired *t* test). AMD = age-related macular degeneration, BCVA = best-corrected visual acuity, logMAR = logarithm of the minimal angle of resolution, NS = not significant, PCV = polypoidal choroidal vasculopathy, RAP = retinal angiomatous proliferation, t-AMD = typical AMD, VA = visual acuity.

In each type of AMD, the mean logMAR BCVA was improved from baseline to 12 months but not significant statistically (Figure 1B).

### Retreatment Rate

The mean number of IVP treatments during the 12-month study period was 5.42 ± 2.30 (range, 2–9).

### CRT Outcomes

**FIGURE 2:**
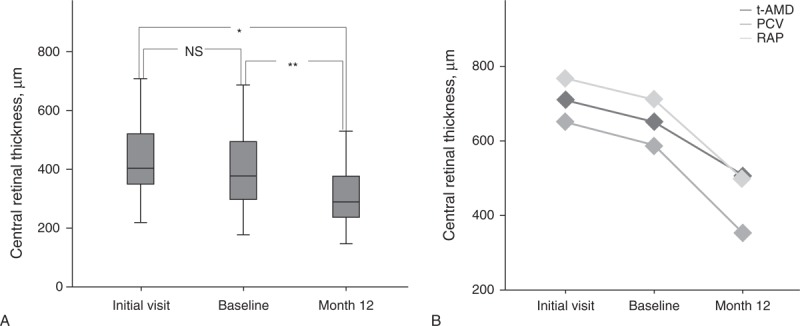
(A) Mean change in CRT (whole type of AMD cases). Box plots show the CRT at the initial visit, baseline, and at 12 months. The CRT (mean ± standard deviation) was 446.9 ± 150.6 μm at the initial visit, 414.5 ± 146.5 μm at baseline, and 317.1 ± 99.1 μm at 12 months. A significant improvement in the CRT was seen between the initial visit and the measurements at 12 months (∗*P* < 0.001) and between the baseline and the measurements at 12 months (∗∗*P* = 0.002) (Bonferroni correction). (B) Mean change in CRT (each type of AMD cases).  
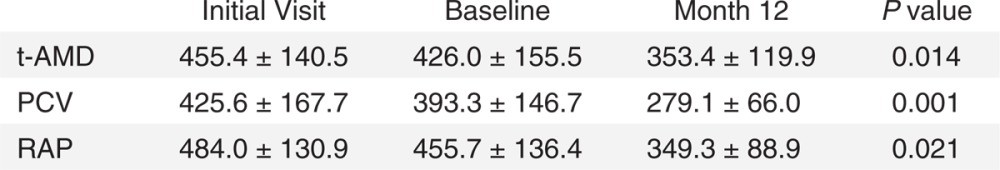

The CRT (mean ± standard deviation) was 446.9 ± 150.6 µm at the initial visit, 414.5 ± 146.7 µm at baseline, and 318.7 ± 100.0 µm at 12 months. Although there was a significant decrease in the CRT at 12 months when compared with the initial visit and baseline measurements (*P *< 0.016, Bonferroni correction), there were no significant differences between the initial visit and baseline values (Figure 2A).

*P* value indicates the statistical correlation of CRT between baseline and 12 months (paired *t* test). AMD = age-related macular degeneration, CRT = central retinal thickness, NS = not significant, PCV = polypoidal choroidal vasculopathy, RAP = retinal angiomatous proliferation, t-AMD = typical AMD.

In each type of AMD, the mean CRT significantly decreased from baseline to 12 months (*P* < 0.05, paired *t* test, Figure 2B).

### Anatomic Improvement

**FIGURE 3:**
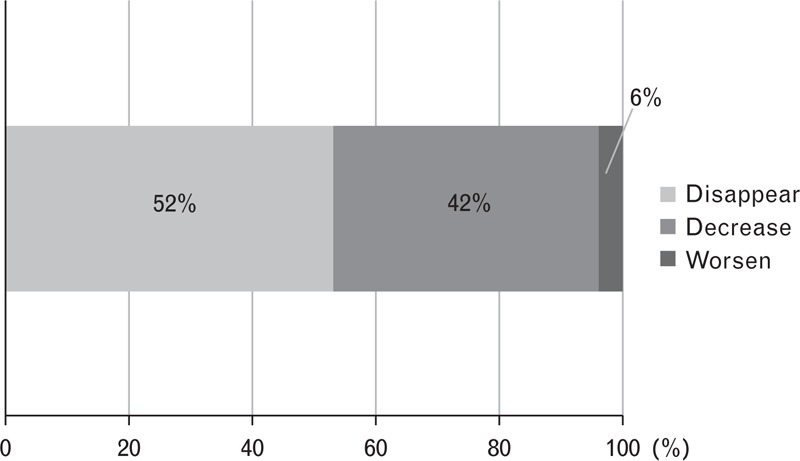
The bar graph shows the change of the exudative lesion from the baseline to 12 months for 50 eyes with AMD, switched from ranibizumab and/or combined PDT to pegaptanib monotherapy. The exudative lesion disappeared completely in 52%, decreased in 42%, and worsened in 6%. AMD = age-related macular degeneration, PDT = photodynamic therapy.

At baseline, cystoid macular edema (CME) was observed in 23 eyes, whereas serous retinal detachment (SRD) was observed in 50 eyes and pigment epithelial detachment (PED) in 29 eyes. The exudative change completely resolved in 27 eyes (52%), decreased in 21 eyes (42%), and worsened in 2 eyes (6%) at 12 months (Figure 3). There was complete resolution of CME in 10 eyes (43.5%), SRD in 41 eyes (82.0%), and PED in 19 eyes (65.5%) at 12 months.

### Factors for Achieving Dry Macula With IVP

Although previous treatment of PDT had no influence on achieving dry macula at 12 months for the patients switched to IVP monotherapy (*P* = 0.39, Mann–Whitney *U* test), there was a significant correlation for the number of IVRs (*P* = 0.014, Mann–Whitney *U* test). Multiple logistic regression analysis revealed that the number of IVRs was significantly associated with achieving dry macula at 12 months (*P* = 0.027), whereas the absence of CME at baseline tended to be associated with dry macula (*P* = 0.064, Table 2).

**Table 2:**
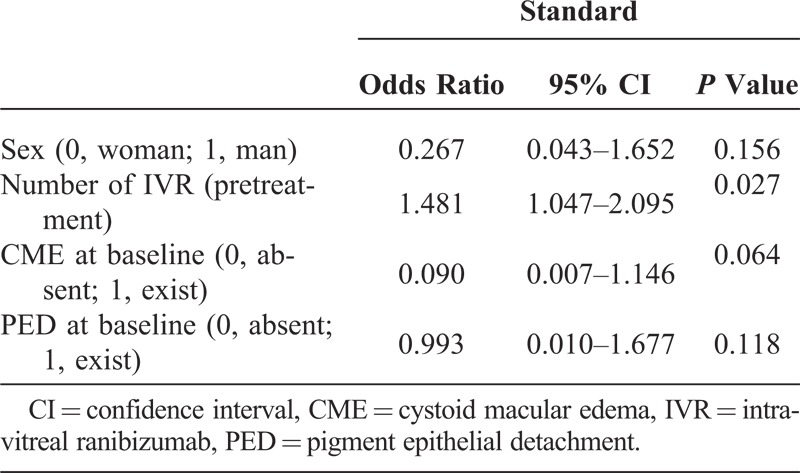
Multiple Logistic Regression Model of Variables Associated With Achieving Dry Macula at 12 Months After Baseline

### Adverse Events and Complications

There were no serious adverse events related to the treatment. In addition, there were no complications such as inflammation, increased intraocular pressure, severe vision loss, endophthalmitis, or systemic thromboembolic events that developed during the study.

### Case Reports

#### Case 1

**FIGURE 4:**
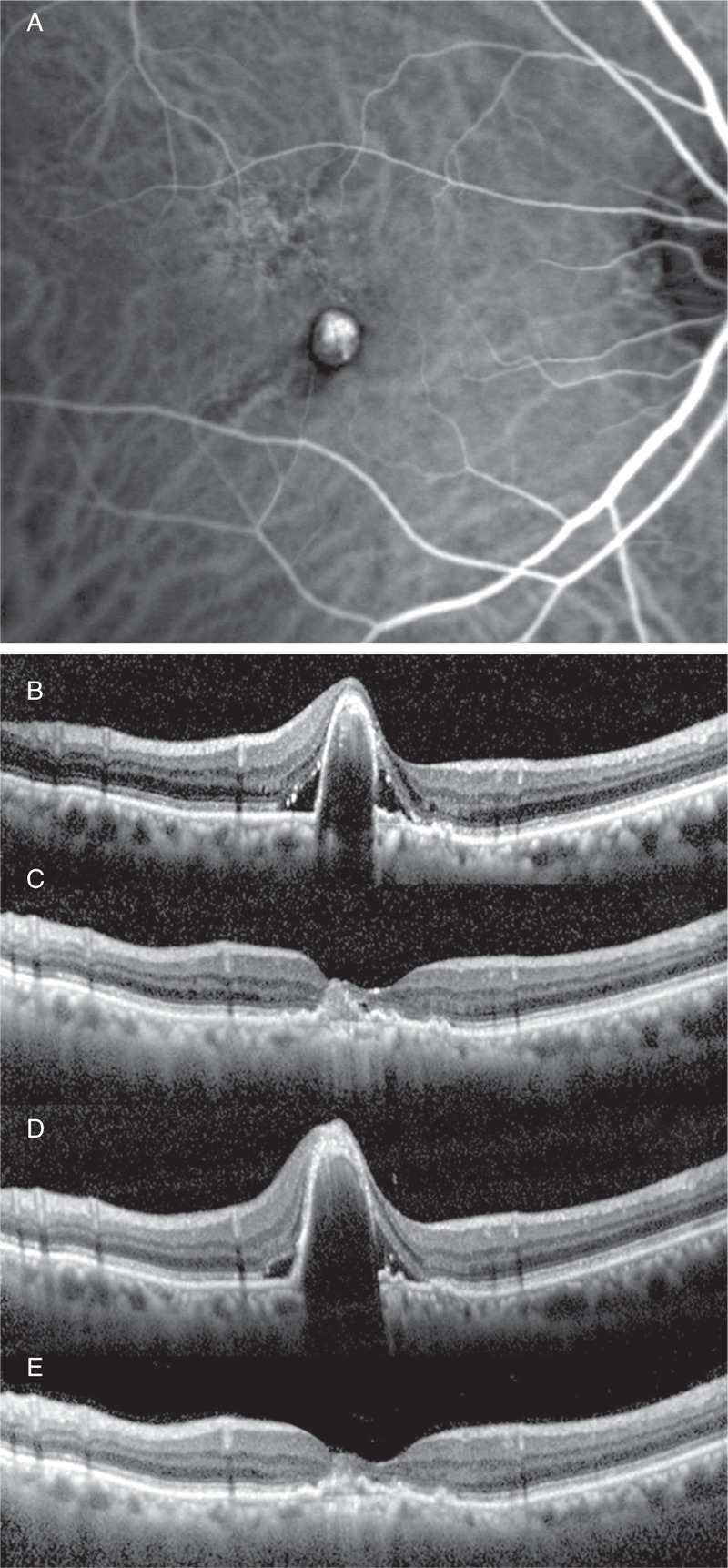
In case 1, the patient was a 74-year-old man with PCV who was resistant to the treatment with ranibizumab-combined PDT. Subfoveal polypoidal lesion and vascular network was showed on early phase ICGA (A). The OCT revealed PED and SRD (B). Although the exudative lesion disappeared after 3 IVR-combined PDT (C), the PED and SRD recurred after 4 additional IVR (D). After that, ranibizumab was switched to pegaptanib; there was complete resolution of the PED and SRD (E). ICGA = indocyanine green angiography, IVR = intravitreal ranibizumab, OCT = optical coherence tomography, PCV = polypoidal choroidal vasculopathy, PDT = photodynamic therapy, PED = pigment epithelial detachment, SRD = serous retinal detachment.

The patient was a 74-year-old man with PCV who was resistant to the treatment with ranibizumab-combined PDT (Figure 4).

At the initial visit, the BCVA was 20/60 in his left eye. Subfoveal polypoidal lesion and vascular network were showed on early phase ICGA (Figure 4A). The OCT revealed PED and SRD (Figure 4B), and the CRT was 407 µm.

Although the exudative lesion disappeared after 3 IVR-combined PDT (Figure 4C), the PED and SRD were increased gradually after 4 additional IVR administrations (Figure 4D). Even though BCVA was maintained at 20/60, the CRT increased to 523 µm at 12 months after the last IVR. Thus, the patient’s anti-VEGF agent was switched from ranibizumab to pegaptanib (baseline).

After the switch, the patient underwent pegaptanib monotherapy 3 times (performed at 6-week intervals). At 12 months after the baseline observations, there was complete resolution of the PED and SRD, BCVA increased to 20/50, and CRT dramatically decreased to 226 µm (Figure 4E).

#### Case 2

**FIGURE 5:**
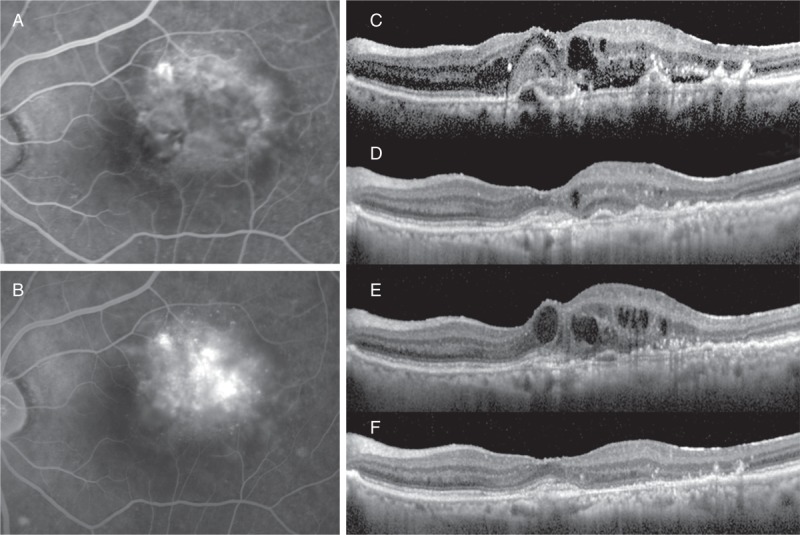
In case 2, the patient was a 90-year-old woman with t-AMD who was resistant to the treatment with ranibizumab monotherapy. The FA showed minimally classic CNV (A, early phase; B, late phase), and OCT revealed the presence of SRD, small PED, and CME (C). The exudative lesion almost resolved after 9 IVRs at 12 months (D). At 3 months after the last IVR, because there was exacerbation of the CME (E), ranibizumab was switched to pegaptanib. At 12 months after baseline, there was complete resolution of the CME (F). AMD = age-related macular degeneration, CNV = choroidal neovascularization, CME = cystoid macular edema, CRT = central retinal thickness, FA = fluorescein angiography, IVR = intravitreal ranibizumab, OCT = optical coherence tomography, RD = subretinal detachment, PED = pigment epithelial detachment, SRD = serous retinal detachment, t-AMD = typical AMD.

The patient was a 90-year-old woman with t-AMD who was resistant to the treatment with ranibizumab monotherapy (Figure 5).

At the initial visit, the BCVA was 20/20 and the CRT was 442 µm in her left eye. FA showed minimally classic CNV (Figure 5A, early phase; Figure 5B, late phase) and OCT revealed the presence of SRD, small PED, and CME (Figure 5C).

The exudative lesion almost resolved after 9 times IVR at 12 months (Figure 5D). Three months after the last IVR, there was exacerbation of the CME, OCT showed the CRT increased to 394 µm (Figure 5E), and her BCVA decreased to 20/40. Thus, the patient’s anti-VEGF agent was switched from ranibizumab to pegaptanib. After the switch, the patient underwent pegaptanib monotherapy 6 times (performed at 6-week intervals). At 12 months after the baseline observations, the CME completely resolved, BCVA improved to 20/20, and the CRT dramatically decreased to 285 µm (Figure 5F).

## DISCUSSION

In the present study, pegaptanib appears to have been strongly effective in those patients who did not respond to frequent IVR and/or multiple PDT therapy. In patients who achieved dry macula at 12 months after switching to pegaptanib, there was a significant association with the number of IVR treatments, but not with the number of PDT procedures. The reason why persistent exudative lesions exist in wet AMD in spite of continued IVR monotherapy and/or combined PDT therapy may be because of the tachyphylaxis that occurs with IVR.

A retrospective study showed that anti-VEGF tachyphylaxis appeared in 38% of the AMD patients given bevacizumab, whereas 19% exhibited tachyphylaxis following treatment with ranibizumab.^7^ In a second retrospective review of 976 patients, the rate of tachyphylaxis was found to be 2%.^8^ In our current study, the rate of persistent cases, which may have been because of tachyphylaxis that occurred with the ranibizumab, was 17.0%.

The reason why pegaptanib is effective in patients with AMD who have developed tachyphylaxis to repeated ranibizumab administrations may be because of the impact of intraocular VEGF_165_, in particular, on the pathological condition. During the initial period when the drug continued to be effective, ranibizumab may have adequately inhibited VEGF-A and been able to block or slow the pathological angiogenesis. Thus, the main stalk cells of CNV may be almost completely occluded by the VEGF_121_ blockade caused by ranibizumab. However, if its ability to completely suppress VEGF-A wanes due to drug tolerance, the VEG_F165_ blockade of pegaptanib may be sufficient by itself to affect the induction of the remaining tip cells of CNV. Given the fact that pegaptanib proved to be effective in the present cases, we conjectured that VEGF_165_, in particular, was able to affect the patient’s condition. In fact, animal experiments and studies in humans have demonstrated the importance of VEGF_165_, which is both soluble and insoluble.^13–16^ In angiogenesis, both soluble and insoluble isoforms are known to be necessary for sprouting. Because the amount of VEGF_165_ is significantly greater than that seen for other VEGF isoforms, including VEGF_121_, this isoform is thought to play a central role in angiogenesis. Thus, this may explain why pegaptanib, which only inhibits VEGF_165_, is effective for ranibizumab-resistant AMD. Moreover, the fact that pegaptanib is immunologically lenient because it is an aptamer and not an antibody might also be a factor in its action.

The other reason why pegaptanib is effective in AMD patients who developed tolerance and stopped responding to repeated administrations of ranibizumab may be because of the growth factor, platelet-derived growth factor (PDGF). This growth factor is involved in a variety of different mechanisms of action, such as being responsible for pericyte recruitment and survival.^17,18^ Furthermore, pericytes confer anti-VEGF resistance.^19^ Because Pfizer’s internal data have shown that pegaptanib has a weak binding ability with PDGF, if pericytes are the source of resistance for the anti-VEGF therapy in neovascular AMD, this suggests there would be a better result when anti-PDGF and anti-VEGF therapies are combined.

Support of this postulate comes from the 2012 American Academy of Ophthalmology Annual Meeting at which phase 2b study results for anti-PDGF and anti-VEGF combination therapy were presented. Although this is just 1 theory, these results suggest that the anti-PDGF effect of pegaptanib could be beneficial for treating patients exhibiting ranibizumab tolerance.

Overall, these previous results suggest that pegaptanib, which targets VEGF_165_ and PDGF, can be of benefit when switching from ranibizumab. Additionally, in patients who show evidence of tachyphylaxis following treatment with ranibizumab for AMD, pegaptanib might be a better alternative to either bevacizumab or aflibercept, which bind to all VEGF-A isoforms.

“Angiogenic” events include sprouting morphogenesis and cell growth, which involves splitting, remodeling, and stabilization.^19–22^ At the cellular level, angiogenesis involves at least 2 distinct cell types, endothelial cells and supporting cells. In addition, it also requires a number of different cellular functions, such as migration, proliferation, cell survival, differentiation, and specialization. During angiogenesis, VEGF plays a key role in most, if not all, of these morphogenic events.

The cellular mechanisms that guide the pattern for vascular sprouting include the graded distribution of VEGF and the sequential steps that involve the induction of a tip cell by VEGF, polarization of a tip cell with rapid directed migration, and proliferation of the stalk cells. The proliferation of the stalk cells is primarily associated with VEGF_121_, whereas the induction and polarization of a tip cell^15,16^ are strongly associated with VEGF_165_.

Although the issue of VEGF-dependent ocular homeostasis is yet to be examined clinically, preclinical data have suggested that during ischemic conditions, VEGF_121_ may play an essential retinal neuroprotective role.^22^ This previous study additionally determined that VEGF_121_, which is the isoform spared by pegaptanib, was required for neuroprotection. When there was sustained inhibition of all of the VEGF-A isoforms, this ultimately led to a progressive loss of the retinal ganglion cells. Moreover, it has been proposed that the pan-VEGF blockade, especially the VEGF_121_ blockade, may be responsible for increasing the GA for AMD, thereby resulting in a poor visual prognosis.^23–26^ In a recent report, that evaluated the 7-year outcomes for ranibizumab-treated patients in ANCHOR, MARINA, and HORIZON databases, macular atrophy was detected by fundus autofluorescence in 98% of the eyes, with a mean area^26^ of 9.4 mm^2^. This report determined that the area of atrophy was significantly correlated with poor visual outcome (*P* < 0.001). The development of macular atrophy leading to GA may be related to the frequency of pan-VEGF-A blockade treatments.^23,24,26^

In this study, the patient group was not homogeneous, both AMD and PCV patients were included. In spite of this difference, similar treatment effect was archived for any type of AMD.

In conclusion, switching patients from ranibizumab to pegaptanib may be able to help maintain or improve the visual function by preventing recurrence of CNV activity in AMD patients who develop tolerance to ranibizumab. The most beneficial properties of pegaptanib use appear to be the maintenance of normal retinal function and the prevention of GA, as pegaptanib does not cause pan-VEGF-A blockage. Larger and long-term observational studies will need to be undertaken in order to definitively determine the efficacy of switching anti-VEGF therapeutic agents in patients with AMD.
